# Supplementary material for: CHC22 clathrin recruitment to the early secretory pathway requires two-site interaction with SNX5 and p115
Source: EMBO J. 2024 Aug 19;43(19):8. doi: 10.1038/s44318-024-00198-y (PMC11445476; doi:10.1038/s44318-024-00198-y)
Supplement: Supplementary file 12 — Expanded View Figures [file 44318_2024_198_MOESM12_ESM.pdf]

Expanded View Figures

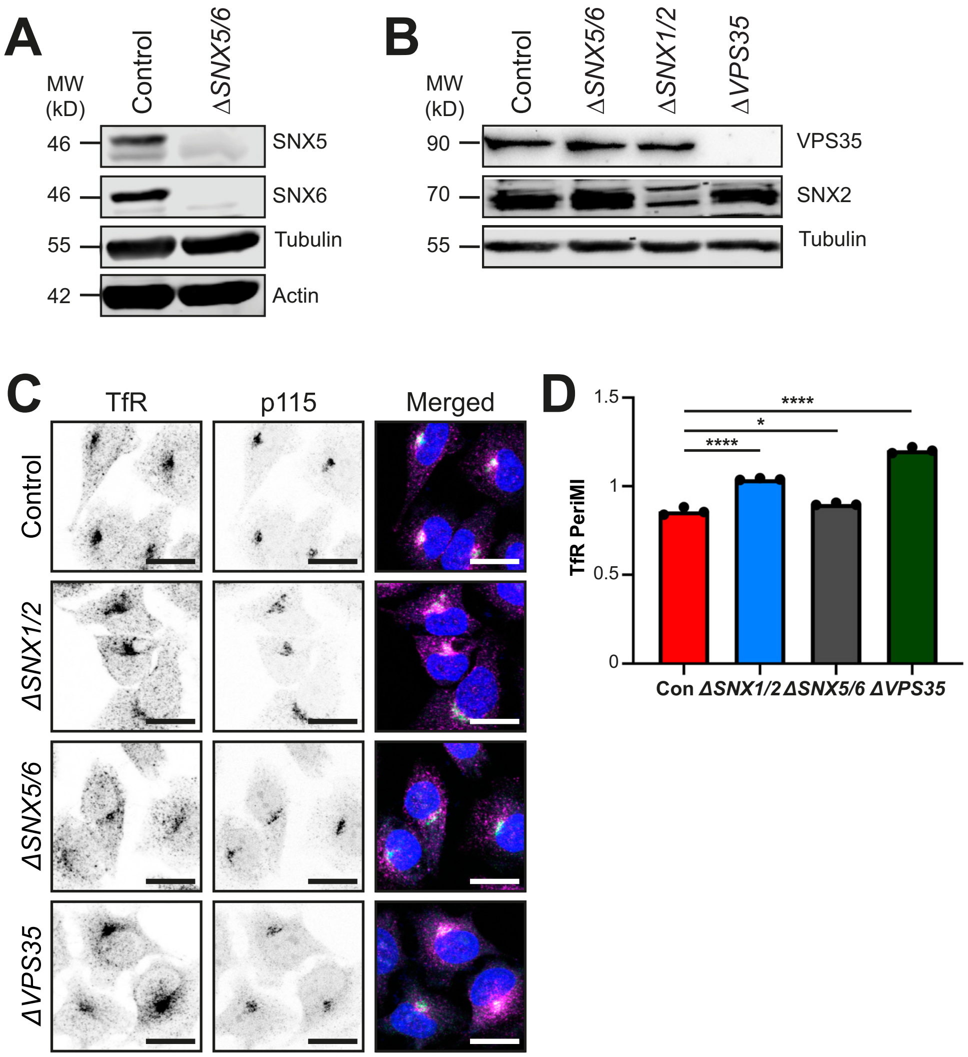

Figure EV1. Phenotype validation of knock-out HeLa cell lines used in the study and the distribution of transferrin receptor (TfR).

(**A**) Representative immunoblot ($n = 2$) of lysates from parental HeLa cells (Control, left) and CRISPR knock-out SNX5/6 HeLa cells (*ΔSNX5/6*, right) immunoblotted for SNX5, SNX6, tubulin, and actin. The migration positions of MW markers are indicated at the left in kilodaltons (kD). (**B**) Immunoblot of lysates from parental HeLa (Control, left) and CRISPR knock-out cell lines; SNX5/6, SNX1/2, and VPS35 (*ΔSNX5/6, ΔSNX1/2, ΔVPS35*) immunoblotted for SNX2, VPS35, and tubulin. The migration positions of MW markers are indicated at the left in kD. (**C**) Representative images of parental HeLa line (top), *ΔSNX1/2* HeLa-derived line (second row), *ΔSNX5/6* HeLa-derived line (third row), and *ΔVPS35* HeLa-derived line (bottom) immunolabeled for TfR (magenta in merged) and p115 (green in merged) with label overlap in white and DAPI-stained nuclei (blue) in merged. Scale bars: 25 µm. (**D**) Quantification of PeriMI for TfR in the HeLa lines shown in (**A**). Each bar represents the mean normalized PeriMI value from three independent experiments, each individually depicted as dots (12–23 cells per genotype, per experiment). Statistical analysis was performed using a one-way ANOVA with a Tukey post-hoc test ($P < 0.0001$(****), $P = 0.0326$ (*)).

© The Author(s)

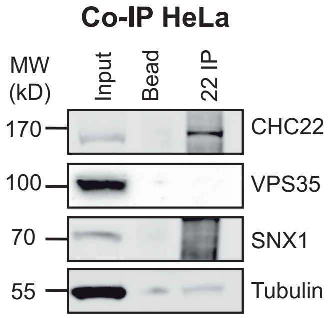

Figure EV2. CHC22 is not complexed with the Retromer and ESCPE-1 components VPS35 and SNX1.

Representative immunoblot ($n = 3$) of CHC22 immunoprecipitate (IP) from HeLa cells. Samples were immunoblotted for CHC22, VPS35, SNX1, and tubulin. Lysate input (5%), bead-only (no antibody) control (Bead) and CHC22 immunoprecipitate (22 IP) are shown. The migration positions of MW markers are indicated at the left in kilodaltons (kD).

 © The Author(s)

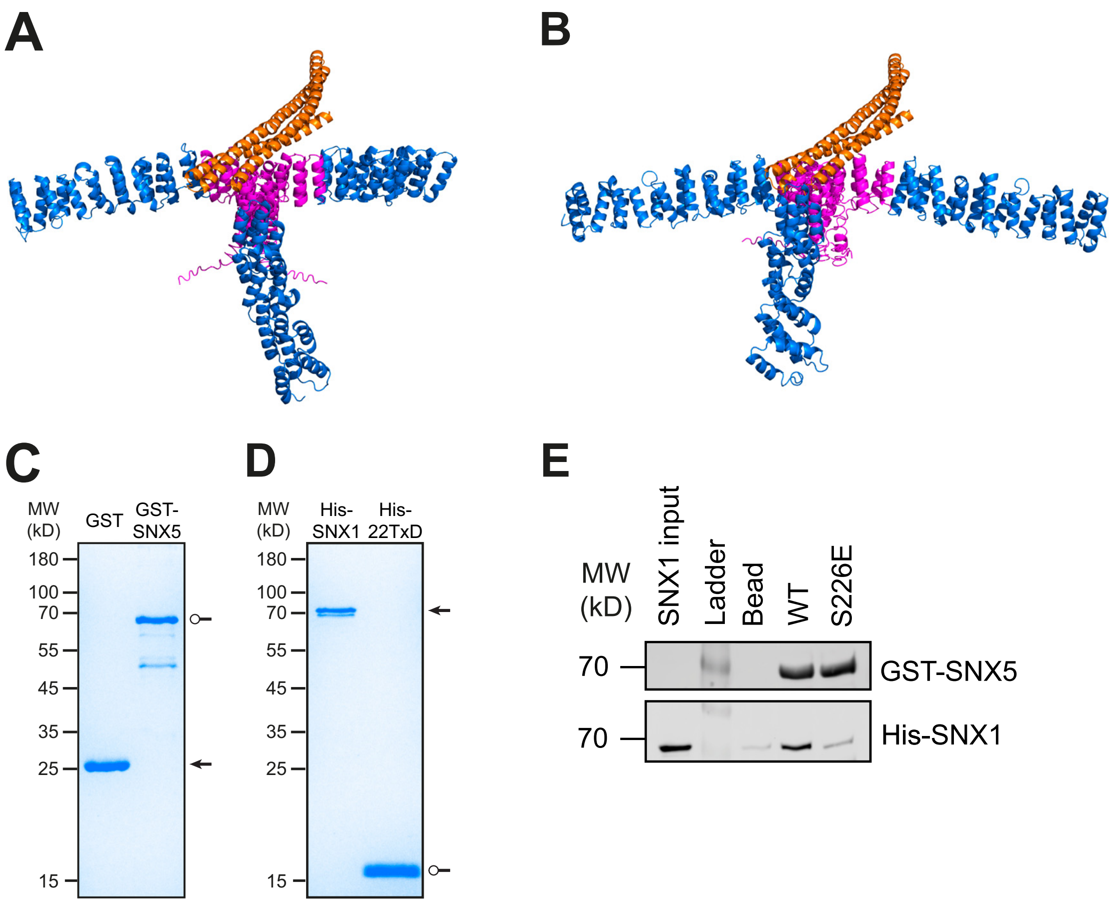

Figure EV3. Binding of SNX6 to CHC22 TxD predicted by AlphaFold and confirmation of SNX5 mutant loss of binding to SNX1.

(**A**, **B**) Comparison of the AlphaFold-generated models of the interaction between CHC22 Hub (blue proximal leg residues 1278–1519 and magenta TxD residues 1520–1640) and the BAR domain of SNX5 (residues 202–404, orange) (A) or SNX6 (residues 205–406, orange) (**B**). (**C**) Coomassie-stained SDS-PAGE gel of GST (arrowhead) and GST-SNX5 (hollow circle) used for in vitro pulldown assays in Figs. 3E,F and 5F. 1 µg protein per lane. (**D**) Coomassie-stained SDS-PAGE gel of His-SNX1 (arrowhead) and His-22TxD (hollow circle) used in the competition binding assay in Fig. 4D. 1 µg protein per lane. (**E**) Representative immunoblot ($n = 2$) of the in vitro binding of purified full-length His-SNX1 to immobilized full-length GST-SNX5 wild-type or full-length GST-SNX5 phosphomimetic S226E mutant. His-SNX1 was added to bead-only control (Bead), GST-SNX5 WT (WT), or GST-SNX5 S226E mutant (S226E). Samples were immunoblotted for GST (top) or the His-tag (bottom) and detected proteins indicated at the right. The positions of MW markers (C-E) are indicated in kD at the left.

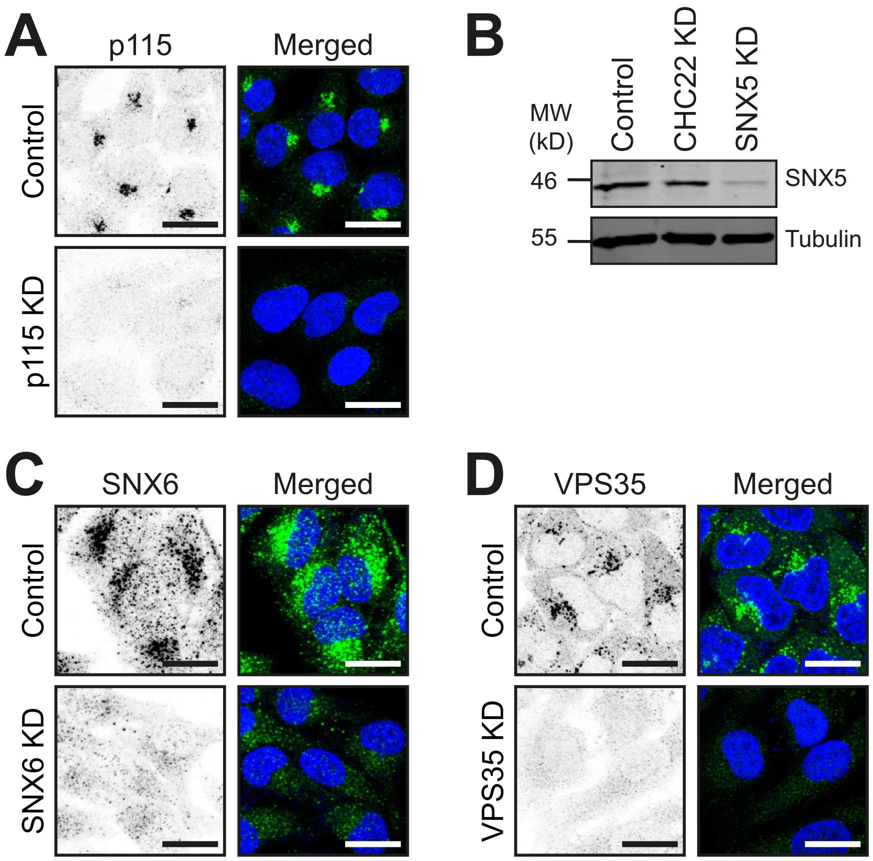

Figure EV4. Protein depletion by siRNA targeting p115, SNX5, SNX6, and VPS35 used in this study.

(**A**) Representative images of HeLa cells transfected with control non-targeting siRNA (Control, top), and cells transfected with siRNA targeting p115 (p115 KD, bottom). Cells were immunolabeled for p115 (green in merged with DAPI-stained nuclei, blue). (**B**) Representative immunoblot of lysates from HeLa cells transfected with control non-targeting siRNA (Control, left) and HeLa cells transfected with siRNA targeting SNX5 (SNX5 KD, left) immunoblotted for SNX5 and tubulin. The migration positions of MW markers are indicated at the left in kD. (**C**) Representative images of HeLa cells transfected with control non-targeting siRNA (Control, top) and cells transfected with siRNA targeting SNX6 (SNX6 KD, bottom). Cells were immunolabeled for SNX6 (green in merged with DAPI-stained nuclei, blue. (**D**) Representative images of HeLa cells transfected with control non-targeting siRNA (Control, top) and cells transfected with siRNA targeting VPS35 (VPS35 KD, bottom). Cells were immunolabeled for VPS35 (green in merged with DAPI-stained nuclei, blue. Scale bars: 25 µm.

 © The Author(s)
